# Durability of Magnesium Potassium Phosphate Cements (MKPCs) under Chemical Attack

**DOI:** 10.3390/ma17174252

**Published:** 2024-08-28

**Authors:** Salma Chhaiba, Sergio Martinez-Sanchez, Nuria Husillos-Rodriguez, Ángel Palomo, Hajime Kinoshita, Inés Garcia-Lodeiro

**Affiliations:** 1 Eduardo Torroja Institute for Construction Science (IETcc-CSIC), 28033 Madrid, Spain; salma.chhaiba@ietcc.csic.es (S.C.); nuria.husillos@ietcc.csic.es (N.H.-R.); palomo@ietcc.csic.es (Á.P.); 2 Department of Material Science and Engineering, University of Sheffield, Sheffield S10 2TN, UK; h.kinoshita@sheffield.ac.uk

**Keywords:** durability, magnesium potassium phosphate cements (MKPCs), K-struvite, low-grade MgO, chemical attack

## Abstract

Magnesium phosphate cements (MPCs), also known as chemically bonded ceramics, represent a class of inorganic cements that have garnered considerable interest in recent years for their exceptional properties and diverse applications in the construction and engineering sectors. However, the development of these cements is relatively recent (they emerged at the beginning of the 20th century), so there are still certain aspects relating to their durability that need to be evaluated. The present work analyses the chemical durability of magnesium potassium phosphate cements (MKPCs) during 1 year of immersion in three leaching media: seawater, a Na_2_SO_4_ solution (4% by mass) and deionized water. For this, pastes of prismatic specimens of MKPC, prepared with different M/P ratio (2 and 3), were submitted to the different chemical attacks. At different ages, the changes on the mechanical strengths, microstructure (BSEM, MIP) and mineralogy (XRD, FTIR, TG/DTG) were evaluated. The results obtained indicate that, in general terms, MKPC systems show good behavior in the three media, with the more resistant system being the one prepared with a M/P molar ratio of 3.

## 1. Introduction

Magnesium phosphate cements (MPCs) also known as chemically bonded ceramics [1], represent a class of inorganic cements that have garnered considerable interest in recent years for their exceptional properties and diverse applications in the construction and engineering sectors. MPCs are formed through a chemical reaction between dead burnt magnesia (MgO) and a phosphate-based source, typically ammonium phosphate (NH_4_) H_2_PO_4_) or potassium phosphate (KH_2_PO_4_). Cements prepared with ammonium dihydrogen phosphate are referred to as magnesium ammonium phosphate cement (MAPC), while those prepared with potassium dihydrogen phosphate are known as magnesium potassium phosphate cement (MKPC) [2,3]. MPCs were originally produced by the reaction of dead burnt magnesia, ammonium phosphate and water [4,5,6,7,8]. The main reaction product obtained was struvite (NH_4_MgPO_4_·6H_2_O). However, ammonium magnesium phosphate cements pose a challenge due to their tendency to release ammonia during mixing and setting (with the consequent health and environmental issues). To address this concern and minimize the environmental impact, magnesium potassium phosphate cement (MKPC) was developed as the next generation of MPCs. By substituting ammonium phosphate for potassium phosphate, MKPC effectively eliminates the ammonia release [9]. The main reaction product in these MKPCs is K-struvite (KMgPO_4_·6H_2_O), which is analog to struvite (NH_4_MgPO_4_·6H_2_O).

In comparison to other cementitious materials, this new type of cementitious system presents distinct and remarkable properties including near-neutral pH, low water demand, low drying shrinkage and high early compressive strengths [10,11,12,13]. One of its key advantages is the rapid strength development at early ages (MKPC mortars can achieve more than 50 MPa in the first 24 h). These high early strengths offer several advantages, making them ideal for rapid repair work, as well as for use in coated materials, biomaterials, and in the preparation of foamed concrete [14,15,16,17].

Similar to conventional cementitious materials, the water-to-binder ratio plays a crucial role in the mechanical properties and microstructure [18,19,20,21]. Furthermore, the magnesia-to-phosphate molar ratio (M/P) significantly impacts both the mechanical and microstructural properties [22]. Le Rouzic et al. [18] investigated the durability of MKPC paste specimens across M/P molar ratios ranging from 1 to 10. They found that a low Mg/P molar ratio (M/P < 5) resulted in residual KH_2_PO_4_, leading to the formation of efflorescences, swelling and cracking. These findings were corroborated by a recent study [22], which specifically identified these problems at M/P molar ratios of 1. However, it has been demonstrated that M/P molar ratios of 3 exhibited good stability over time from a mechanical, mineralogical and microstructural point of view [23].

In terms of durability, which is a crucial factor for assessing a new material, the research remains relatively limited. It has to be noticed that these cements are relatively new (the first studies date from the early 20th century and the literature available is scarce and sometimes contradictory [24]. Jie Shi et al. [25] demonstrated that MKPC paste specimens are more sensitive to sulfuric acid attack than MAPC paste specimens. After being immersed in sulfuric acid (pH 2) for 28 days, MKPC showed a significant decrease in compressive strength due to the amorphization of struvite-K crystals upon sulfuric acid exposure. In contrast, MAPC paste specimens contained a higher amount of struvite and larger struvite crystals, which contributed to their better resistance to sulfuric acid. Jianming Yang’s research [26] shows that after 30 days of seawater soaking, the compressive strength of MKPC paste specimens rises to 68.2 MPa, a 50% increase from its initial strength of 45.4 MPa. Additionally, he found that allowing the MKPC paste specimens to hydrate naturally for 28 days instead of 3 days before seawater immersion improves its resistance to seawater erosion [26].

On the other hand, the microstructure of MPC-based materials can be altered in contact with water. This phenomenon was observed by Yang et al. [24], whose investigation revealed that that magnesium ammonium phosphate mortars (M/P molar ratio of 3 and a w/b ratio of 0.44) experienced a 10% decrease in compressive strength after 28 days of immersion in water and a 20% decrease after 90 days.

Previous research [27,28] found that a prolonged initial air curing time of 28 days improves the water resistance of MKPC paste specimens (60 days of immersion). When immersed in water, MKP dissociates into ions, leading to MgO dissolution and the formation of white crystals on the surface, which slows corrosion in static water. In flowing water, increased dissolution and leaching prevent recrystallization, causing microcracks and weakening bonds, thus reducing the strength and water resistance due to the lack of white crystal formation on the surface.

Considering the limitations mentioned above, and based on the findings of our previous research [23], we have opted to focus on magnesium potassium phosphate cement pastes (MKPCs) that exhibited good performance, with the particularity that the source of MgO used to make these cements has been a low-grade MgO (a secondary product obtained in the calcination of magnesite). Considering all the mentioned above, the main objective of this research was to investigate the mineralogical and microstructural evolution of MKPCs over time (up to 1 year) when subjected to different media: deionized water, a sodium sulfate solution (Na_2_SO_4_) and synthetic seawater.

## 2. Experimental

### 2.1. Materials

A low-grade MgO (L-MgO), provided by MAGNESITAS NAVARRAS S.A (Navarre, Spain), was used as a source of MgO. The mineralogical analysis (XRD, Rietveld Analysis RWp 7.62) revealed that the main crystalline phase was periclase (MgO 57.62%). Table 1 shows the composition in % mass of phases corresponding to the Rietveld analysis, where secondary minor components, such as magnesite (MgCO_3_), dolomite (MgCa(CO_3_)_2_), quartz (SiO_2_), calcite (CaCO_3_) and brucite (Mg(OH)_2_), can be identified. The particle size distribution was measured using a Laser Particle Size Analyzer, Mastersizer 2000, Jinan, China, while the surface area was determined with a Micromeritics Gemini VII. The particle size distribution showed D90, D50, and D10 values of 6.07 μm, 23.2 μm, and 59.90 μm, respectively, and the surface area was 186.06 m²/g. As a source of phosphate, a KH_2_PO_4_ soluble salt (sold as fertilizer) with a purity > 98 mass % (SIGMA ALDRICH, St. Louis, MO, USA) was used. Because the reaction between magnesium oxide and potassium dihydrogen phosphate is extremely rapid, an appropriate quantity of retardant (boric acid (H_3_BO_3_), purity > 99.5 wt. %, SIGMA ALDRICH) was added to delay the setting (otherwise the components react very fast, limiting its workability).

### 2.2. Preparation of Magnesium Phosphate Cements MKPC Pastes

For the preparation of the MKPC pastes, two different M/P molar ratios were used, 2 and 3. The water/binder (w/b) ratio used was 0.24. Boric acid (added as a retardant) was pre-dissolved in water and then the mixture of water + boric acid was added to the binder (low-grade MgO + KH_2_PO_4_). The proportion of boric acid/binder (by mass) was 0.025. Samples were mixed in a rotator at 1300 rpm for 3 min, and then be casted in prismatic molds to produce 1 × 1 × 6 cm^3^ specimens. These specimens were then cured under two curing conditions (CC and LAB), where were maintained up to 28 days:

- CC: In a climatic chamber at 21 ± 3 °C and 99 ± 5% relative humidity (RH).
- LAB: In the laboratory at 21 ± 3 °C and 52 ± 5% of relative humidity (RH).

After these 28 days, four set of samples, for each curing regime, were prepared. One set was used as a reference (and its mechanical strengths, mineralogy and microstructure was determined). The other three sets were submitted to the different type of chemical attacks. For that, paste prismatic specimens were immersed in three different media: deionized water (pH 6.15), a sodium sulfate Na_2_SO_4_ (SIGMA ALDRICH) (4.4% mass) solution (pH 8.4) and a synthetic seawater (pH 8). The specimens were maintained in the different solutions for 1 year. To avoid the supersaturation, the leaching solutions were renewed periodically at the ages of 3, 7, 28, 56, 90 and 180 days. After 28, 90, 180 and 365 days of immersion, the specimens were tested from the mechanical (compressive strengths).

The preparation of the solutions consisted of the following: For the Na_2_SO_4_ solution, the required amount of sodium sulfate was dissolved in water to achieve a concentration of 4.4 g of sulfate per 100 g of solution (4.4% by mass) [20]. The synthetic seawater was prepared according to the standard ASTM 1141-98 [29]. Its compositions is mainly based on NaCl and MgCl_2_ (see Table 2) with minor proportion of sodium sulfate and sodium bi-carbonate.

### 2.3. Experimental Methods

Six replicates per series were used to determine the compressive strengths. Testing was conducted using an Ibertest Autest 200/10-SW (Madrid, Spain) test frame, the average and standard deviation of the results were then calculated and reported.

Prior to microstructural characterization (Mercury Intrusion Porosimetry (MIP) and Scanning Electron Microscopy with Energy Dispersive X-ray Analysis (BSEM/EDX), one of the specimens, left unbroken, is submitted to immersion in isopropanol for 2 days to stop further hydration reactions. Subsequently, they were dried in a desiccator for a minimum of 48 h to eliminate any residual isopropanol. To prepare the powder samples (for the mineralogical analysis), specimen fragments were ground to pass through a 63 µm sieve as per previous recommendations to stop the reaction processes. Subsequently, they were mixed with isopropanol for 3 min, filtered and placed in a vacuum desiccator until a constant weight was attained. Changes in the pore structure were evaluated by mercury intrusion porosimetry (MIP) on a Micromeritics Poresize 9320 IV.09 mercury intrusion porosimeter (Micromeritics Instrument Corporation, Norcross, GA, USA), assuming a sample–mercury contact angle of 140°. The microstructure of the samples was studied by Backscattering Electron Microscopy (BSEM) on a JEOL JSM6400 scanning electron microscope (Tokyo, Japan). Additionally, a semi-quantitative analysis of the chemical composition of the reaction products was conducted via Energy Dispersive X-ray spectroscopy (EDX) on a Links ISIS EDX analyzer, collecting at least 40 points from the cementitious matrix per sample; the data were processed using the Bruker ESPRIT 1.9 software, whereas the mineralogical and chemical composition of samples were studied via X-ray Diffraction (XRD), Fourier Transform Infrared Spectroscopy (FTIR) and Thermogravimetric analysis (TG/DTG).

XRD measurements were carried out on a Bruker D8 Advance diffractometer (Karlsruhe, Germany) in a 2θ range of 5–60° with a step size of 0.02° every 0.5 s using CuKα radiation at 40 kV and 30 mA. The existing phases were identified and quantified using the DiffracPlus EVA 4 2.1 and TOPAS 5.0 software, in conjunction with a chemical reconciliation. A Nicolet 6700 spectrometer (Thermo Fisher Scientific, Waltham, MA, USA, 02451) was employed for FTIR analysis, covering a range of 400 cm^−1^ to 4000 cm^−1^ with a resolution of 4 cm^−1^, using powdered samples embedded in KBr pellets (0.001 g sample/0.099 g KBr). Thermogravimetric analysis (TGA) was conducted using a Perkin-Elmer TG analyzer (PerkinElmer, Shelton, USA). Powdered samples underwent heating from room temperature to 800 °C at a rate of 10 °C min^−1^ under a N_2_ flow of 200 cm^3^/min.

## 3. Results and Discussion

### 3.1. Mechanical Strengths

**Figure 1:**
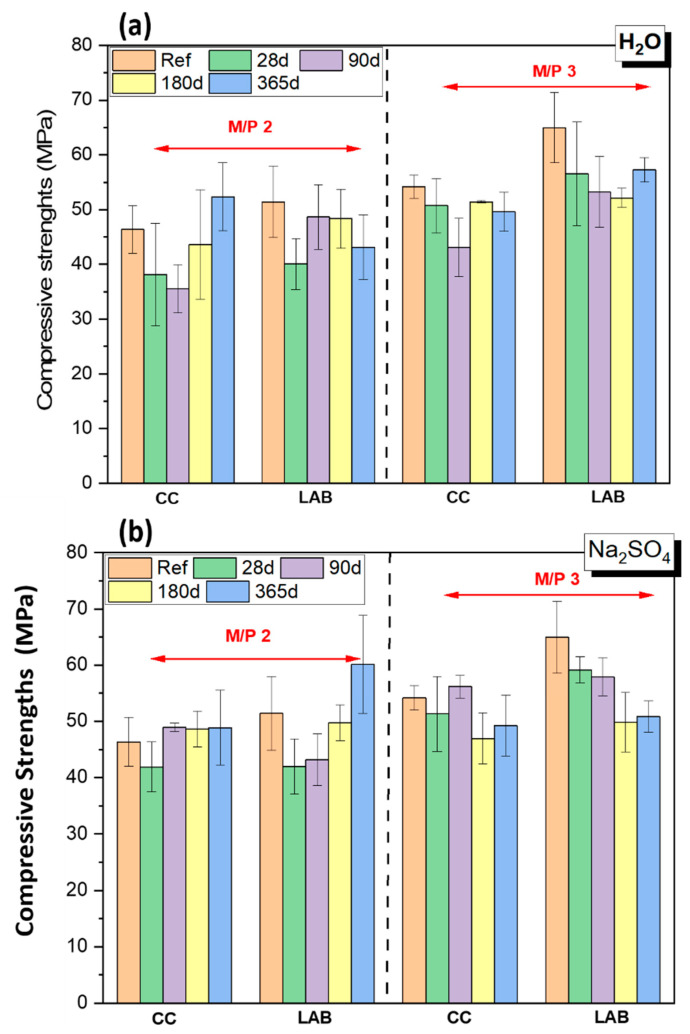

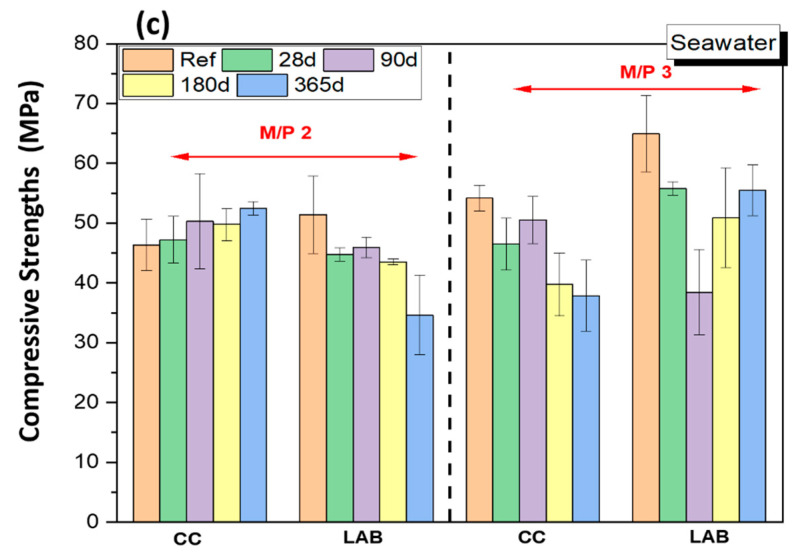
Compressive strengths evolution of MKPCs under solution attacks (**a**) H_2_O; (**b**) Na_2_SO_4_; (**c**) seawater (samples immersed during 1 year).

Figure 1 illustrates the variation on compressive strengths of MKPC samples at different ages before (Reference sample) and after the immersion (28, 90, 180 and 365 d) in the different solutions. The resistance to water and chemical solutions is a crucial aspect of MKPC durability.

Before the immersion (reference samples), the data indicate a notable enhancement in the compressive strength, particularly when the M/P molar ratio was 3, under laboratory curing conditions (LAB) (the strengths overpass 65 MPa after 28 d of curing), indicating that curing conditions, especially relative humidity, also play a substantial role in the mechanical strength development.

The strengths increased from 51.7 MPa in the MKPC systems designed with a M/P ratio of 2 to 65.14 MPa for those prepared with a M/P 3, which confirm that the M/P molar ratio also plays a substantial role in the strengths. However, if the M/P ratio is too high [23], there may not be enough phosphate to react with all the MgO, resulting in the formation of fewer reaction products and poor cohesion between the binding phase and unreacted particles. Conversely, low M/P ratios can lead to an excess of phosphates, which may leach out of the matrix, causing changes in the microstructure over time [23,30].

The samples after the immersion show different behavior. In general terms, there is a decline in the strengths with respect to the reference samples, regardless of the type solutions in which they were immersed. It is also observed that MKPC systems prepared with M/P 3 behaves better than those prepared with a M/P ratio of 2.

Figure 1a illustrates that the compressive strength of both M/P ratios 2 and 3 decreases over time after immersion in deionized water, regardless of the initial curing conditions. However, it has to be considered that the error bars are too high to draw definitive conclusions on the behavior, although for both M/P ratios and both curing conditions, a slight decrease in the strength values is observed.

Regarding the samples immersed in the Na_2_SO_4_ solution (see Figure 1b), their compressive strength showed sensitivity to the sulfate chemical attack, especially for samples cured in the laboratory where the decline in the strengths is more evident. For paste specimens cured in a climatic chamber, a decrease in compressive strength occurred at 28 days of immersion, followed by an increase at 90 days, maintaining this value until one year for M/P ratio 2. However, for M/P ratio 3, a decrease was observed with prolonged sodium sulfate immersion.

Samples cured in laboratory conditions exhibited a decrease in compressive strength after immersion for M/P ratio 2, followed by an increase that surpassed the initial reference value before immersion, reaching its maximum at one year. However, for M/P ratio 3, their compressive strength was significantly lower than the reference after immersion in sodium sulfate, with no increase observed over time, reaching its minimum value at one year.

Figure 1c presents the compressive strength of samples after seawater immersion. Except for those with an M/P ratio of 2 and cured in the climatic chamber (CC), all samples show a decrease in strength, this decrease is more significant in samples cured in the laboratory.

The behavior of the different systems will be further explored in the sections on mineralogical and microstructural characterization.

### 3.2. Changes in the Porosity after the Chemical Attack

**Figure 2:**
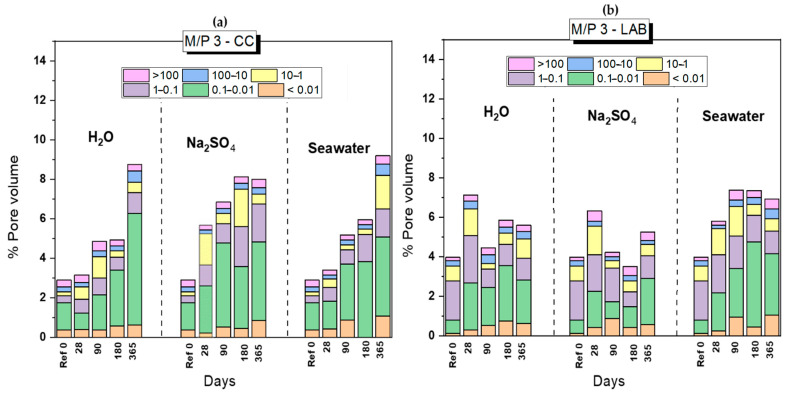
Total porosity and pore size distribution of MKPC paste specimens (M/P 3) cured in (**a**) climatic chamber (CC) and (**b**) laboratory (LAB), before and after the chemical attack in deionized water (H_2_O), seawater and the Na_2_SO_4_ solution.

Based on the previous results, MKPC systems prepared with a M/P ratio of 3, were selected, since they present higher mechanical strengths, to explore changes in the porosity and the consequences of the chemical attack. Figure 2 shows the changes over time (from 28 to 1-year f immersion) on the total porosity and in the pore size distribution of MKPCS systems, cured in both conditions (climatic chamber and laboratory), before and after the chemical attacks (H_2_O, Na_2_SO_4_ and seawater). In all cases, there is an increase in total porosity, with respect to the reference material, which would justify the decrease in mechanical strengths observed in Figure 1. It has also been observed that those cements cured in the climatic chamber (CC, 21 °C, 99% RH) undergo higher porosity increase than those cured in the laboratory (LAB, 21 °C, 52% RH). In the first case, regardless of the type of attack (H_2_O, Na_2_SO_4_ or Seawater), the total porosities after 1 year of immersion are very similar (around 9–10%), with values that practically triple the value of the reference system (around 3% of total porosity). As expected, the total porosity increases with the increasing of immersion time.

It is also observed that the immersion processes in the different media have a clear effect on the pore size distribution, increasing the proportion of larger pores, especially in the 0.1–1 μm, 1–10 μm and 10–100 μm intervals, which is another factor that justifies the decrease in the strengths. These increases in total porosity and modifications in the pore size ranges were also observed by Jueshi Qian.

At selected ages (28, 90, 180 and 365 days), MKPC samples (prepared with an M/P ratio of 3 and cured in the CC) were extracted from the different media (H_2_O, Na_2_SO_4_ and seawater). These samples were mineralogically analyzed by XRD, FTIR and TG/DTG, and their microstructure was further examined using BSEM/EDX.

### 3.3. Mineralogical Analysis

**Figure 3:**
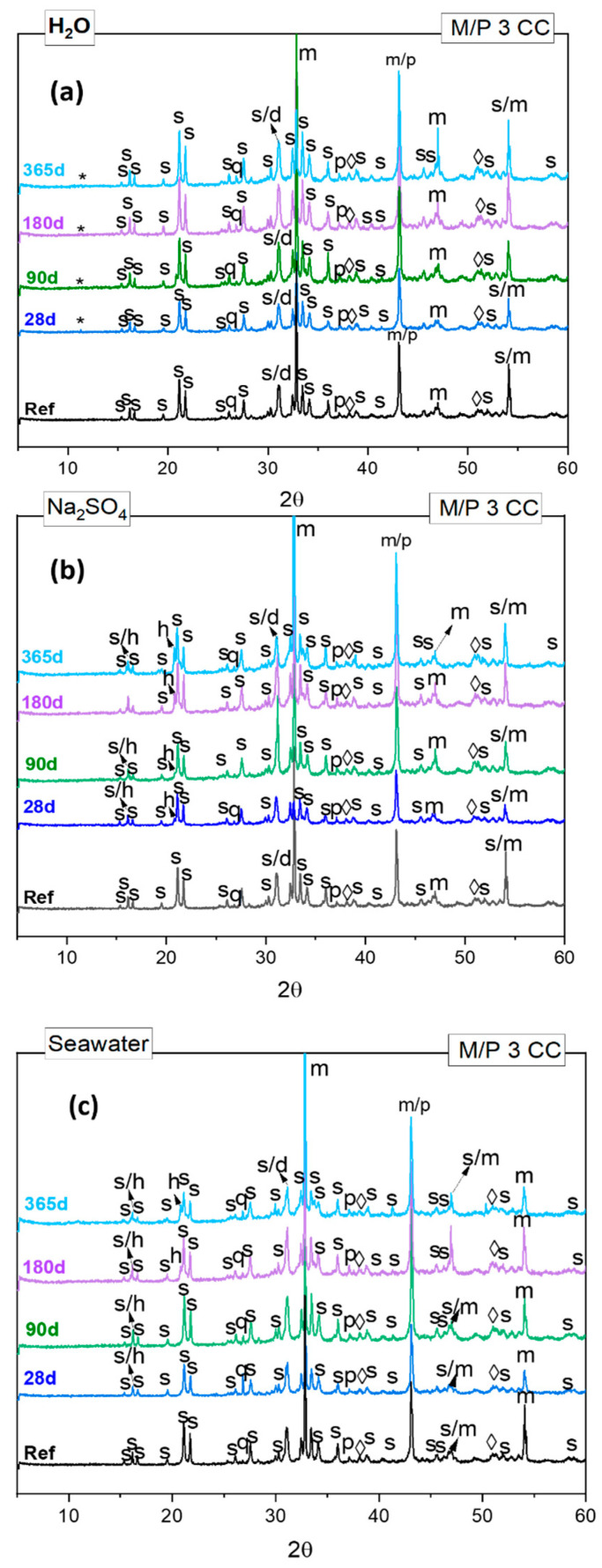
XRD patterns of MKPC samples with M/P ratios 3 cured in the climatic chamber and exposed to (**a**) water, (**b**) Na_2_SO_4_ and (**c**) seawater attack solutions at 7, 28, 90, 180 and 365 days. (Legend: S: K-struvite (MgKPO_4_·6H_2_O-COD: 9011199), P: Periclase (MgO-COD: 9007058), m: magnesite (MgCO_3_-COD: 9000096), d: Dolomite (CaMg(CO_3_)_2_-COD: 1200014), q: Quartz (SiO_2_), **◊**: Brucite (Mg(OH)_2_-COD: 9006330) h: Hazenite ((KNaMg_2_(PO_4_)_2_·14H_2_O)-COD: 9012213), *: cattiite (Mg_3_(PO_4_)_2_·22 H_2_O-COD: 9008294)).

Figure 3 shows the XRD patterns corresponding to reference samples, along with those corresponding with paste specimens immersed in different solutions after 1 year. In all the systems, regardless the immersion media, the main reaction product identified was the K-struvite (MgKPO_4_·6H_2_O).

Peaks corresponding to unreacted particles of periclase (MgO) and magnesite (MgCO_3_) were also detected. Other minor phases, present in the low-grade MgO (such as, dolomite calcite, quartz, …, etc.) were also identified, indicating that are not really affected by the different immersion media.

Apart from these, some other crystalline phases were observed. Brucite (Mg(OH)_2_) was detected in the reference and after 28 days in all samples, appearing with a very small intensity (2 Theta = 37.97 and 50.8). We have to consider that a minor proportion of brucite was also identified in the low-grade MgO, probably due to the reaction between MgO and the atmospheric humidity during the storage. Furthermore, no differences were observed between the samples at 28 days and those after 1 year, regardless of the solution attack used.

After 28 days, samples immersed in deionized water, show the formation of a new crystalline magnesium phosphate hydrate, cattiite Mg_3_(PO_4_)_2_⋅22H_2_O, identified at 2 Theta = 11.08, which was not present in the reference system. Early studies developed by Taylor et al. and Wei et al. [31,32] found that K-struvite, in the presence of deionized water, will be partially converted into cattiite (Equation (1)):

3MgKPO_4_⋅6H_2_O (K-struvite) + 16H_2_O → Mg_3_(PO_4_)_2_⋅22H_2_O (cattiite) + 3K^+^ + PO_4_ ^3−^ (1)

This partial conversion could lead to low the strengths of MKPC according to Biwan Xu et al. [33].

At same age (28 d), in samples immersed in sulfate and seawater solutions, hazenite (KNaMg_2_(PO_4_)_2_⋅14H_2_O) was identified as a new crystalline phase Its intensity slightly increases over time, suggesting that hazenite could have precipitated due to the partial dissolution of K-struvite [34,35] when materials are immersed in these different chemical media. Actually, the presence of Na^+^ (coming from the sodium sulfate and seawater solutions) tends to destabilize K-struvite, leading to the formation of hazenite. In other words, in our cementitious systems, Na^+^, HPO_4_^2−^ and Mg^2+^ species are present in the pore solution, which can react with the main reaction product formed: K-struvite. Under certain conditions, such as high pH (pH 9.5) and the presence of these elements, hazenite precipitates, and H^+^ ions are produced. This phenomena was already described by Yang et al. (See Equation (2)) [36].

2KMgPO_4_⋅6H_2_O + Na^+^ + 8H_2_O +HPO_4_^−^ + Mg^2+^ → KNaMg_2_(PO_4_)_2_⋅14H_2_O↓ + H^+^ (2)

**Figure 4:**
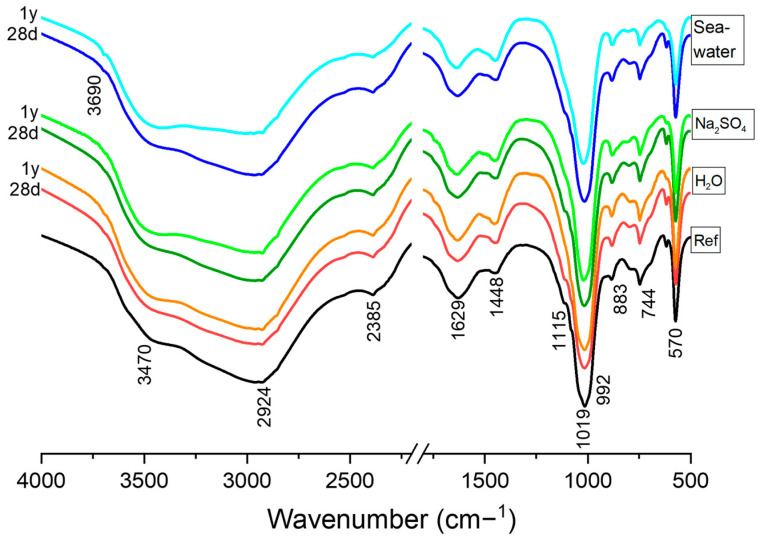
FTIR spectra of M/P 3 samples cured in climatic chamber (CC) before (Ref) and after 28 day and 1 year solution attacks (H_2_O, Na_2_SO_4_ and seawater).

Considering the previous results, samples after 28 and 365 days of immersion were selected for further analysis. Figure 4 shows the FTIR spectra for MKPC sample (M/P 3) after the immersion in the three media (deionized water, Na_2_SO_4_ and seawater). In the same figure is plotted the FTIR spectrum corresponding to the reference system (MKPC sample before the immersion test). All the spectra show practically the same bands, except the samples immersed in deionized water and seawater, which showed a small sharp peak at 3690 cm^−1^, attributed to the asymmetrical stretching bonds of O-H in brucite (Mg(OH)_2_).

Most of the detected bands are associated with different vibration modes of K-struvite [37]. The absorption bands observed at 3470 cm^−1^ and 2924 cm^−1^ are attributed to the stretching vibrations of H–O–H in water of crystallization. Weak bands at 2385 cm^−1^ can be assigned to the stretching vibrations of H–O–H in clusters of water molecules of crystallization. A medium intensity band at 1630 cm^−1^ indicates bending modes of H–O–H vibrations, suggesting the presence of water from K-struvite. Additionally, a medium absorption band at 883 cm^−1^ indicates the wagging modes of vibration of coordinated water.

The symmetric stretching vibration (ν1) of PO_4_ units corresponding to K-struvite was observed in the range of 950–1023 cm^−1^ and at the peak 570 cm^−1^ [37]. Regarding the cattiite (Mg_3_(PO_4_)_2_·22H_2_O) and hazenite (KNaMg_2_(PO_4_)_2_·14H_2_O) identified in XRD, they can share some similar absorption bands related to the phosphate groups (PO_4_) and water molecules (H_2_O) as K-struvite, since the three of them are magnesium phosphate hydrates [38] and thus overlapping phenomena can occur. Generally, K-struvite may exhibit additional or shifted bands due to the presence of potassium ions, which are absent in cattiite. Additionally, the intensity and exact positions of water-related bands will differ due to varying hydration levels (six water molecules in K-struvite vs. twenty-two in cattiite). Since XRD showed a very small cattiite intensity, Fourier Transform Infrared Spectroscopy (FTIR) may only detect small absorbance bands of cattiite, making it challenging to distinguish the mentioned differences.

Additionally, minor signals associated with different vibration modes of magnesite, dolomite, anhydrite, and quartz were also detected [39,40]. Bands located between 1440 and 1450 cm^−1^ and at 744 cm^−1^ are attributed to the C-O asymmetric stretching band, corresponding to magnesite and dolomite. Furthermore, the band at 1115 cm^−1^ can be assigned to symmetrical stretching vibration of Si–O bonds in quartz.

**Figure 5:**
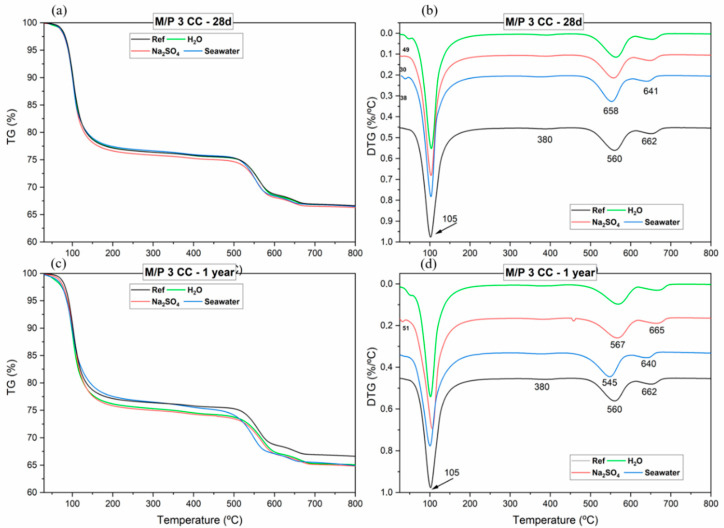
Thermogravimetric curves: (**a**,**c**) TG and (**b**,**d**) DTG curves of MKPC (M/P 3, and cured in CC) for reference systems and the attacked samples (H_2_O, Na_2_SO_4_ and seawater) at 28 days and 1 year.

Figure 5 shows the TG (a) and (c) and DTG (b) and (d) curves for the samples prepared with an M/P ratio of 3, cured in climatic chamber and subjected to deionized water, sulfate and seawater solution attacks after 28 days and 1 year. The same graph includes the TG/DTG curves corresponding to the reference system. All TG/DTG curves show similar trends, with two main mass loss events: the first occurring between 50 and 200 °C, showing a large mass loss peak center around about 105 °C, that according to the literature, is characteristic of the dehydration temperature for K-struvite [41]. The mass loss of the K-struvite (See Equation (3)), which is the dominant phase in all MKPC systems, begins at around 50 °C and ends at around 250 °C [14,28]:

MgKPO_4_⋅6H_2_O → MgKPO_4_ + 6 H_2_O↑ (3)

Moreover, cattiite (Mg_3_(PO_4_)_2_·22H_2_O) and hazenite (KNaMg_2_(PO_4_)_2_·14H_2_O) can exhibit a weight loss at around 100 °C, similar to K-struvite (KMgPO_4_·6H_2_O). The dehydration processes of both compounds occur within similar temperature ranges, typically due to the loss of water from these hydrated phases [41].

The second weight loss, located between 480 and 670 °C, corresponds to the decarbonation of magnesite and dolomite, present as minor components in the low-grade MgO [23]. The small peak appearing around 380 °C is attributed to the dehydroxilation of brucite (Mg(OH)_2_) (this phase was already detected by X-ray Diffraction (XRD) and Fourier Transform Infrared Spectroscopy (FTIR)).

Table 3 shows the mass loss corresponding to the different intervals for all samples. The total mass loss, in all systems, was about 36% of the original mass. The mass loss associated with the K-struvite (or other hydrated phosphate phases) dehydration, exposed to chemical attacks, showed slight differences compared to the reference sample. Specifically, the systems exposed to deionized water and Na_2_SO_4_ for 1 year show slightly higher loss (24.17% and 24.69%, respectively) than the reference (23.35%). The system exposed to seawater for 1 year, shows, however, a slightly lower (23.11%) loss than the reference.

Brucite showed a slight increase in weight loss compared to the reference, mainly after 1 year of exposure, indicating that part of periclase in the low-grade MgO is reacting with water to form Mg(OH)_2_ during the immersion test. Despite the formation of brucite, no sign of any expansion or deterioration was observed in the specimens.

### 3.4. Microstructural Analysis

**Figure 6:**
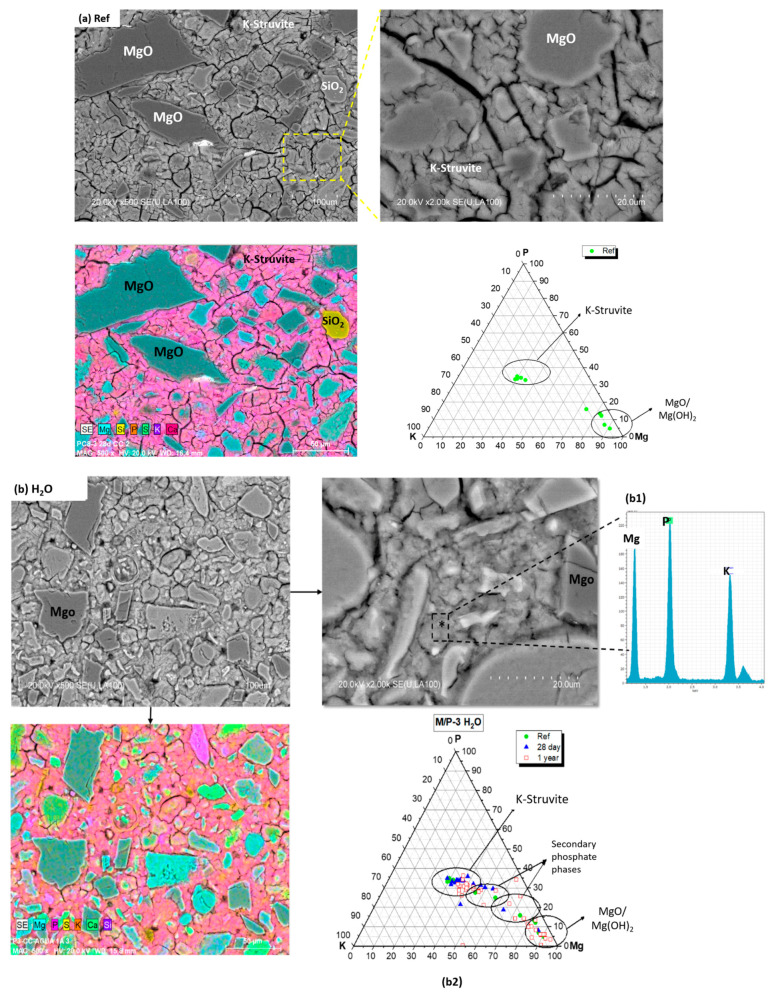

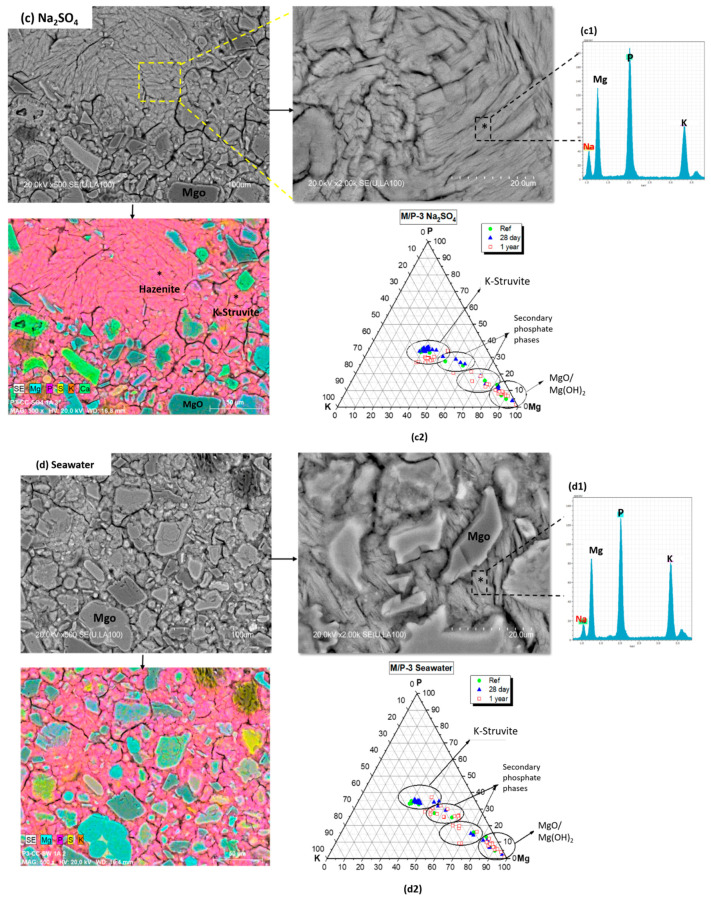
Backscattering Electron Microscopy (BSEM) micrographs and mappings of the reference sample (**a**) and samples immersed for 365 days in (**b**) H_2_O, (**c**) Na_2_SO_4_ and (**d**) seawater. Energy Dispersive X-ray Analysis (EDX) (**b1**, **c1** and **d1**) and plotted in the Mg-P-K ternary diagram (**b2**, **c2** and **d2**) after 28 and 365 days of immersion (green points correspond with reference system, blue points after 28 d immersion and red points after 1 year).

Figure 6a shows the micrographs and EDX mapping corresponding to the reference system before the immersion. Figure 6a–c shows, respectively, the BSEM morphology, as well as EDX analysis (plotted in the ternary diagram P-Mg-K) for the reference system and the systems after 28 days and 1 year of immersion in the three solution attacks (H_2_O, Na_2_SO_4_ and seawater).

All the micrographs (before and after immersion) show the typical morphology described in the literature for MKPC pastes [41,42]. It can be observed that the presence of a significant number of unreacted periclase (MgO) particles are surrounded by a cementitious matrix, which have a chemical composition based on Mg, P and K (EDX-b1) attributed to K-struvite crystals. Secondary crystalline phases present in the low-grade MgO (as identified in the X-ray Diffraction analysis), such as quartz, magnesite, dolomite and anhydrite, were also observed in the mappings. The ternary diagram (P-Mg-K) shows that for the reference systems, almost all compositions analyses (more than 30 analysis) were located around the theoretical K-struvite (Mg/P = 1, Mg/K = 1, K/P = 1). The cluster of composition close to the Mg corner is associated with unreacted periclase.

After 1 year immersion in the three solutions, BSEM micrographs revealed some microstructural changes in the K-struvite phase over time associated with a partially loss of its crystallinity [25]. This may result from the partial dissolution–precipitation of K-struvite in the solution attack.

Energy Dispersive X-ray Analysis (EDX) for samples immersed in Na_2_SO_4_ and seawater (EDX-c1 and EDX-d1) show the presence of sodium, together with Mg, K and P, that can be attributed to the formation of hazenite (Mg_2_KNa(PO_4_)_2_·14H_2_O) following immersion in Na_2_SO_4_ and seawater, as identified by X-ray Diffraction. This suggests that sodium ions (Na^+^) can partially replace K^+^ ions (via ion exchange mechanism), causing some type of distortion leading to the partial dissolution of K-struvite and the subsequent precipitation of hazenite [43]. Generally, in the presence of other cations such as sodium, new minerals like hazenite and cattiite can form. In the case of cattiite, the presence of sodium ions can increase the solubility of K-struvite, leading to its dissolution. This dissolution releases magnesium and phosphate ions into the solution, making them available for the formation of cattiite [44]. These processes change the crystal structure and composition of the initial K-struvite, and this is confirmed by the EDX results in the ternary diagram, showing the formation of secondary phosphate phases (cattiite and hazenite).

**Figure 7:**
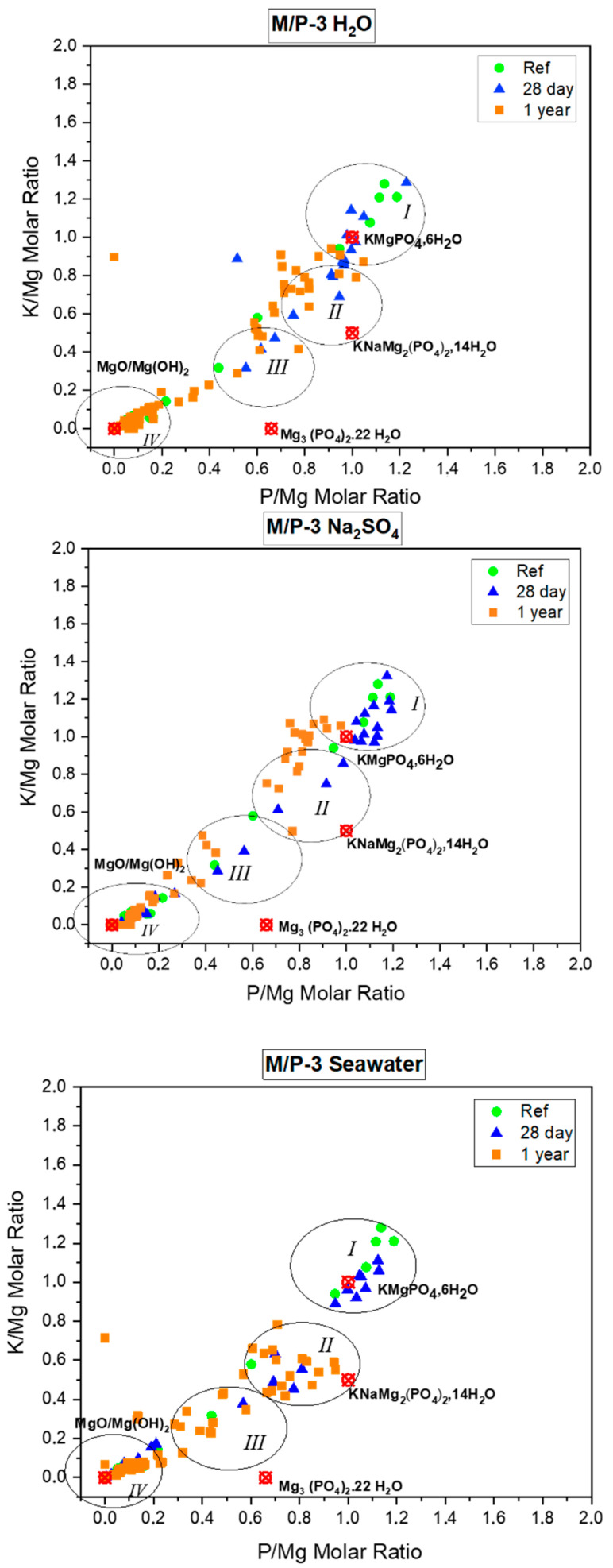
Compositional clusters (I, II, II and IV) analyzed by EDX and associated with the different phases detected by BSEM in the MKPC systems (M/P 3 cured in the climatic chamber) in the reference systems and in after the chemical attacks (28 and 365 days in water, Na_2_SO_4_ and seawater), compared with the theoretical phases (red symbol).

Figure 7 shows the evolution over time of the composition clusters (EDX analysis) of the different phases detected in the MKPCs in the different leaching media. In the same figure, the theoretical composition of the crystalline phases identified in the XRD patterns has been marked: MgO, Mg(OH)_2_, Mg_3_(PO_4_)_2_·22H_2_O, NaKMg_2_(PO_4_)_2_·14H_2_O and K-struvite (MgKPO_4_·6H_2_O). Four clusters of composition can be distinguished:

(I) A cluster of compositions close to the theoretical K-struvite (P/Mg = 1 and K/Mg = 1), showing higher P/Mg ratios and possibly higher K/Mg ratios. These compositions are observed in the reference samples and those immersed in solution for 28 days. According to the literature [30], the higher P/Mg ratio (with respect to the theoretical P/Mg = 1) observed in the reference and immersed samples at 28 days could be explained by several reasons: (i) The initial formulation of the system has a higher value than the theoretical P/Mg ratio of 1. (ii) Phosphate anions could be adsorbed on the crystal surface disguising the EDX analysis, or (iii) other phosphates formed with the impurities originally present in the unreacted magnesia. Notably, after one year of immersion, these compositions shits towards a lower K/Mg ratio, particularly in samples immersed in seawater, suggesting a partial dissolution of this phase (which could be associated with a certain amorphization process observed in the BSEM). This suggests that the compositions close to the theoretical K-struvite are more stable in the short term (28 days) but tend to disappear after long-term immersion (1 year), particularly in seawater environments.
(II) A cluster close to the theoretical hazenite (NaKMg_2_(PO_4_)_2_·14H_2_O). This cluster is observed in samples immersed in seawater and sodium sulfate solutions, but with lower P/Mg molar ratios than the theoretical value. This suggests that these samples tend to form hazenite-like phases under these conditions, though with a slightly altered stoichiometry.
(III) A cluster close to the chemical composition of cattiite (Mg_3_(PO_4_)_2_·22H_2_O): This cluster is found in samples immersed in deionized water, especially at 1 year. However, these compositions exhibit a higher K/Mg molar ratio, implying that some K^+^ ions could be adsorbed on the surface, which results in the detection of a small proportion of potassium in the EDX analysis.
(IV) A cluster with a chemical composition close to MgO and brucite Mg(OH)_2_. With respect to this cluster, we have detected the presence of brucite in small proportion, especially in samples after 1 year of immersion (in the three leaching media) as confirmed by FTIR and TG/DTG analyses. These data points could represent this phase, but with phosphate adsorbed on the surface of magnesium oxide and could affect the surface chemistry and detection in analytical techniques like EDX, leading to a slight deviation in the expected stoichiometry. This phenomenon has also been observed by other researchers [30].

## 4. Conclusions

This paper investigates the durability of magnesium potassium phosphate cements (MKPCs) under chemical attack, focusing on the effects of deionized water, sodium sulfate and seawater. The experimental results above explained provide detailed information for better understanding of the mineralogy and microstructural behavior of the MKPC before and after immersion in those chemical media. Based on these observations, the following conclusions could be drawn:

1. The M/P ratio and the curing conditions affects the microstructure of the cementitious systems and therefore their durability.
2. Chemical attacks in water, Na_2_SO_4_ solution and seawater negatively affect the mechanical strengths and increase the total porosity of MKPC paste specimens; however, compressive strengths remain above 40 MPa and no significant damage was observed. The system with an M/P molar ratio of 3 demonstrated superior mechanical strengths.
3. Regardless of the type of the immersion solution, the main reaction product was K-struvite. Unreacted particles (periclase MgO, magnesite, quartz and dolomite) do not seem to be altered by the chemical solutions after 1 year of immersion.
4. After immersion in the three solution media, the paste specimens exhibited slight amorphization of the K-struvite crystals, which is more evident after 1 year of attack (suggesting their partial instability over long-term immersion). The partial dissolution of the K-struvite, together with the presence of ionic species in the medium favor to the formation of secondary reaction products such as hazenite and cattiite.
5. The presence of brucite was observed, especially after 1 year of immersion; however, this phase appears in very small proportions suggesting a low degree of hydration of the unreacted MgO particles (likely to be adsorbed on the surface of magnesium oxide).

## Figures and Tables

**Table 1:** Composition of the low-grade magnesia (L-MgO) in mass % analyzed using Rietveld methodology.

L-MgO	Periclase (MgO)	Magnesite (MgCO_3_)	Dolomite (CaCO_3_)	Brucite (Mg(OH)_2_)	Quartz (SiO_2_)	Anhidrite (CaSO_4_)	Calcite (CaCO_3_)	RWp
(%)	57.62	25.77	5.41	3.12	1.96	3.97	0.70	7.62

**Table 2:** Composition of synthetic seawater.

Compound *	Concentration (g/L)
NaCl	24.53
MgCl_2_	5.2
Na_2_SO_4_	4.09
CaCl_2_	1.16
KCl	0.69
NaHCO_3_	0.201
KBr	0.101
H_2_BO_3_	0.027
SrCl_2_	0.025
NaF	0.003

* *All copund are from Sigma-Aldrich*.

**Table 3:** Mass loss (%) corresponding to loss of water of hydrated phosphates and brucite.

	Mass Loss (%)			
	Leaching Solutions	Interval 30–200 °C ^(1)^	Interval from 300 to 380 °C ^(2)^	Total Loss
Reference		23.35	0.8	35.67
MKPC 28 days	H_2_O	23.32	1.13	35.84
	Na_2_SO_4_	23.88	1.18	36.34
	Seawater	23.09	0.93	36.01
MKPC 1 year	H_2_O	24.17	1.45	36.27
	Na_2_SO_4_	24.69	1.3	36.87
	Seawater	23.11	2.0	36.58

^(1)^ Loss of water in hydrated phosphates; ^(2)^ dehydroxilation of brucite.

## Data Availability

The original contributions presented in the study are included in the article, further inquiries can be directed to the corresponding author.
